# Expanding the Spectral Responsivity of Photodetectors via the Integration of CdSe/ZnS Quantum Dots and MEH−PPV Polymer Composite

**DOI:** 10.3390/polym16162371

**Published:** 2024-08-21

**Authors:** Thanh Thao Tran, Ha Trang Nguyen, Ankush Sharma, Young-Bin Cho, Manjeet Kumar, Ju-Hyung Yun

**Affiliations:** 1 Department of Electrical Engineering, Incheon National University, Incheon 406772, Republic of Korea; thaotran@inu.ac.kr (T.T.T.); trangnh@inu.ac.kr (H.T.N.); ankush@inu.ac.kr (A.S.); binswon@ktr.or.kr (Y.-B.C.); 2 Water Environmental Analysis Center, Korea Testing & Research Institute (KTR), Gwacheon 13810, Republic of Korea

**Keywords:** organic–inorganic nanocomposite, polymer, quantum dots, photodetector

## Abstract

This study investigates the energy transfer mechanism between the organic polymer poly(2-methoxy-5(2’-ethyl)heroxyphenylenevinylene) (MEH−PPV) and CdSe/ZnS core-shell quantum dots (CdSe/ZnS CSQDs). Additionally, a hybrid ZnO-based photodetector (PD) is fabricated using the composite of MEH−PPV and CdSe/ZnS CSQDs, aiming to gain deeper insights. The combination of MEH−PPV and CdSe/ZnS CSQDs facilitates a broad spectral response in PDs, spanning from the ultraviolet (UV) to the visible range. In particular, PDs with QDs in the composite demonstrate notably excellent photosensitivity to both ultraviolet (UV) light (365 nm) (~5 fold) and visible light (505 nm) (~3 fold).

## 1. Introduction

ZnO holds significant promise for photodetector (PD) applications [1,2,3]; however, a limitation lies in its absorption primarily within the UV spectrum, restricting its utility in broader optical sensing applications. To extend the ZnO optical absorption range into the visible spectrum, other materials with high absorbance in the visible region, such as nanoparticles, polymers, or composite structures, have been incorporated. Recently, organic–inorganic nanocomposites have found wide applications in optical and electronic fields. Wang et al. published a pioneering report on the photoconductivity of CdS QDs and a N-polyvinyl carbazole (PVK) composite [4]. Jia et al. introduced a facile and efficient approach to fabricate zero-dimensional (0D) covalent organic frame network (COF) quantum dots (COQDs) by selectively disassembling hydrogen–covalent hybridized organic frameworks (H–COFs) [5]. Chen et al. were also successful in fabricating highly sensitive visible PDs by mixing CdTe QDs with P3HT/PCBM polymers [6]. Conjugated polymers have attracted considerable attention in photonics and optoelectronic applications [7,8]. Among them, the MEH−PPV polymer is widely chosen as a component in active layers due to its significant absorbance within the visible spectrum. Ghasemi et al. presented the impact of a nanofiller (Al_2_O_3_) in the polymer matrix MEH−PPV on optical, optoelectronic, and electrical properties [9]. Malik et al. fabricated a PD based on MEH:PPV:VOPvPhO:CdSe that showed a fast response and recovery times (~1.25 and 2 s) [10]. Moreover, Chikalova-Luzina et al. conducted a theoretical analysis of the optical properties of a hybrid optoelectronic structure composed of MEH:PPV and CsPbBr3 [11]. Also, Ayesha et al. successfully investigated field-effect transistors (FETs) based on MEH:PPV and a CsPbBr_3_ composite [12]. 

CdSe/ZnS QDs are considered as effective inorganic semiconductor materials for photo-electrochemical applications due to their high absorption coefficients, and high chemical and thermal stability [13]. For instance, Kim et al. devised a promising deposition technique aimed at fabricating controllable layer-by-layer (LbL) structures of CdSe/ZnS QDs, to enhance PD performance [14]. In addition, Vu et al. explored the influence of CdSe/ZnS QDs on the defect states and carrier transport in methylammonium (MA) lead halide perovskites, leading to an improvement in photoresponsivity [15]. The utilization of CdSe/ZnS QDs in photodetector applications represents a promising avenue for advancing optical sensing technologies. 

In this study, an organic–inorganic composite is created by incorporating CdSe/ZnS CSQDs into the MEH−PPV polymer. Subsequently, a PD based on the MEH−PPV:CdSe/ZnS CSQD composite is constructed and analyzed to further enhance comprehension. CdSe/ZnS QDs can facilitate better charge separation due to their high surface area and the formation of a type-II heterojunction with MEH−PPV. This can reduce recombination losses and improve charge transport [16,17]. The QDs can act as electron acceptors (during charge transfer process) [18], enhancing the separation of photo-generated excitons and contributing to a higher photocurrent. The Förster resonance energy transfer (FRET) from CdSe/ZnS QDs to MEH−PPV polymer molecules was also investigated. This process allows the efficient transfer of excitation energy from the QDs, which act as energy donors, to the polymer, serving as the energy acceptor. As a result, the FRET mechanism improves the generation and separation of charge carriers, leading to an increased photocurrent and an overall enhanced device efficiency [19]. MEH−PPV has a broad absorption spectrum, with a peak typically around 500–550 nm, making it efficient for absorbing visible light [20]. CdSe/ZnS QDs have size-tunable absorption and emission properties [21], allowing for complementary absorption to MEH−PPV. Also, CdSe and ZnS quantum dots exhibit high absorbance in the UV range [22]. The integration of both MEH−PPV and CdSe/ZnS CSQDs in the PD also expands its detection capability to encompass both UV and visible wavelengths, thus achieving a broader range of detection. Hence, hybrid organic–inorganic photodetectors represent a logical choice for combining the unique properties of inorganic QDs and organic polymers.

## 2. Materials and Methods

Materials: All chemical reagents, including MEH−PPV polymers, cadmium oxide (CdO, 99.99%), zinc acetate (Zn(CH_3_CO_2_)_2_, 99.99%), selenium powder (Se, 99.5%), sulfur powder (S, 99.5%), tri-n-octylphosphine (TOP, 97%), oleic acid (OA, 90%), 1-octadecene (ODE, 90%) and toluene (99.8%), were purchased from Sigma-Aldrich (Burlington, MA, USA). Zinc oxide (ZnO, 99.99%) was purchased from iTasco (Seoul, Korea). 

Synthesis of CdSe/ZnS CSQDs: The TOP-Se stock solution was prepared through the dissolution of Se (0.2 mmol) and S (4 mmol) in TOP. Subsequently, CdO (0.4 mmol) and Zn(CH_3_CO_2_)_2_ (4 mmol) were dissolved in OA in a flask. This mixture was then gradually heated to 155 °C for 20 min. Following this, a swift injection of 15 mL of ODE into the flask occurred, and the temperature was further raised to 320 °C until the solution turned clear. The resulting transparent solution contained Cd(OA)_2_ and Zn(OA)_2_. For the formation of CdSe/ZnS core-shell quantum dots (QDs), the previously prepared Se-stock solution was introduced into the solution containing Cd(OA)_2_ and Zn(OA)_2_. Similarly, the prepared TOP-Se stock solution was injected into the transparent solution to yield CdSe/ZnS core-shell QDs. The QDs were purified using a hexane/methanol extraction process. QDs with a size of ~6.5 nm (see Figure S1 in ESI) with green emission (Green QDs) and ~9 nm with red emission (Red QDs) were obtained. 

**Figure 1:**
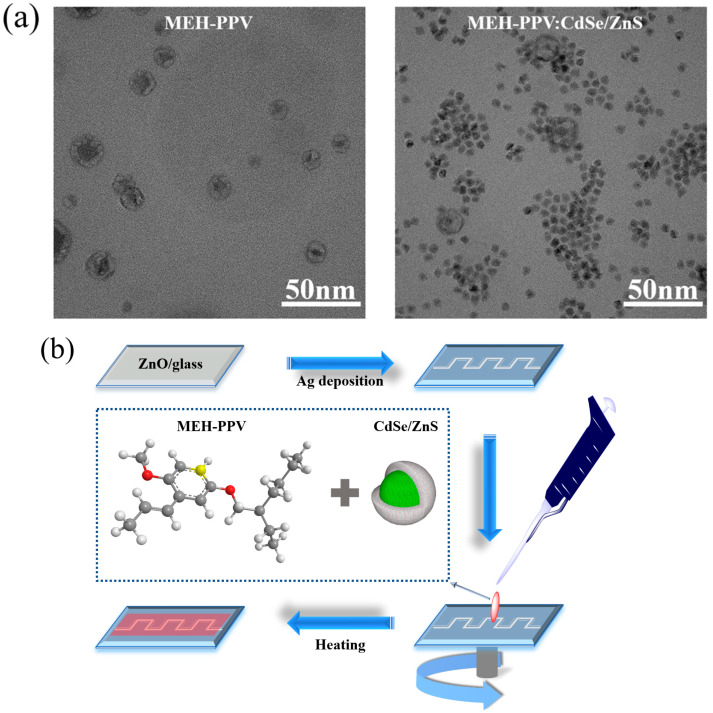
(**a**) Transmission electron microscope (TEM) images of MEH−PPV and MEH−PPV:CdSe/ZnS composites (QDs concentration is 5%), and (**b**) schematic diagram of MEH−PPV:CdSe/ZnS-based PD.

Composite of MEH−PPV:CdSe/ZnS CSQD preparation: MEH−PPV was dissolved in toluene at a concentration of 10 mg/mL. The solution was filtered through a 0.45 µm filter to remove any impurities or undissolved particles. The MEH−PPV:CdSe/ZnS composites were prepared by mixing MEH−PPV and the CdSe/ZnS CSQD solution. The weight ratio of QDs in the mixtures was 3%, 5% (Figure 1a) and 10%, respectively.

Device fabrication: The schematic diagram of the fabricated PD is shown in Figure 1b. A glass substrate was cleaned with acetone and isopropyl alcohol, respectively; finally, it was irradiated by UV-O_3_ treatment to eliminate all the organic impurities and improve the wettability of the substrate. A ZnO thin film (150 nm) (see Figure S2 in ESI) was deposited using RF magnetron sputtering in an Ar gas flow of 50 sccm at a power of 300 W and at room temperature. The silver (Ag, 99.9%) source and drain were deposited by a thermal evaporator under the pressure of ~5 × 10^−6^ torr at room temperature. The composites in the MEH−PPV:CdSe/ZnS CSQD solution dissolved in toluene were spin-coated onto a ZnO-on-glass substrate at 3000 rpm in 1 min, followed by a heating step at 60 °C for 20 min. The thickness of the MEH−PPV:CdSe/ZnS was ~95 nm (see Figure S2 in ESI).The gap of the active area was maintained at 0.2 mm. 

CdSe/ZnS CSQDs and MEH−PPV:CdSe/ZnS CSQD thin film characterizations.: The morphological investigation was performed using a scanning field emission electron microscope (FE−SEM, JEOL JSM-7001F, 15 kV) and transmission electron microscopy (FE-TEM, Thermo Fisher Scientific Talos F200X, 200 kV) analysis. A UV-Vis spectrophotometer (EMCLAB, EMC-11-UV spectrometer) was employed to record the optical absorption spectra of the films. The photoluminescence (PL) measurement was carried out by the Time-Correlated Single Photon Counting (TCSPC) system (Model FluoTime300/MicroTime100). The electrical measurements were characterized using the Keithley 4200-SCS system. The light source was controlled by a BioLED Light Source Control Module (BLS-Series). The intensity of the light sources is provided in Table S1 (supplied in ESI).

## 3. Results and Discussions

### 3.1. Absorbance Properties of MEH−PPV:CdSe/ZnS Fim

**Figure 2:**
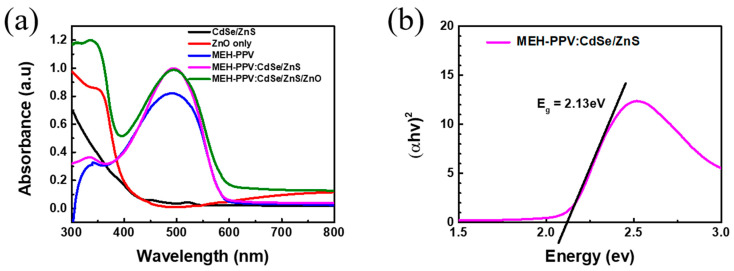
(**a**) Absorbance spectrum and (**b**) optical bandgap using Tauc’s plot of the MEH−PPV:CdSe/ZnS mixture. The concentration of CdSe/ZnS in the composite is 5%.

Figure 2a illustrates the UV-vis absorbance spectrum of various thin films. Remarkably, both CdSe/ZnS CSQDs and ZnO thin films exhibit exceptional absorbance in the UV region, while the MEH−PPV thin film displays outstanding absorbance in the visible region. Interestingly, the combination of MEH−PPV:CdSe/ZnS/ZnO demonstrates high absorbance across a wide spectrum of wavelengths. This feature makes it well-suited for use in broad-spectrum PDs.

The optical bandgap was plotted by using Tauc’s plot. For direct bandgap materials, the relationship between the optical bandgap and photon energy is given in Equation (S1) (provided in ESI). The extrapolation of the straight-line portion of (αhν)^2^ versus (hν) for the MEH−PPV:CdSe/ZnS CSQDs revealed that the optical bandgap of the nanocomposites is E_g_ = 2.13 eV (Figure 2b).

### 3.2. Energy Transfer between CdSe/ZnS and MEH−PPV

**Figure 3:**
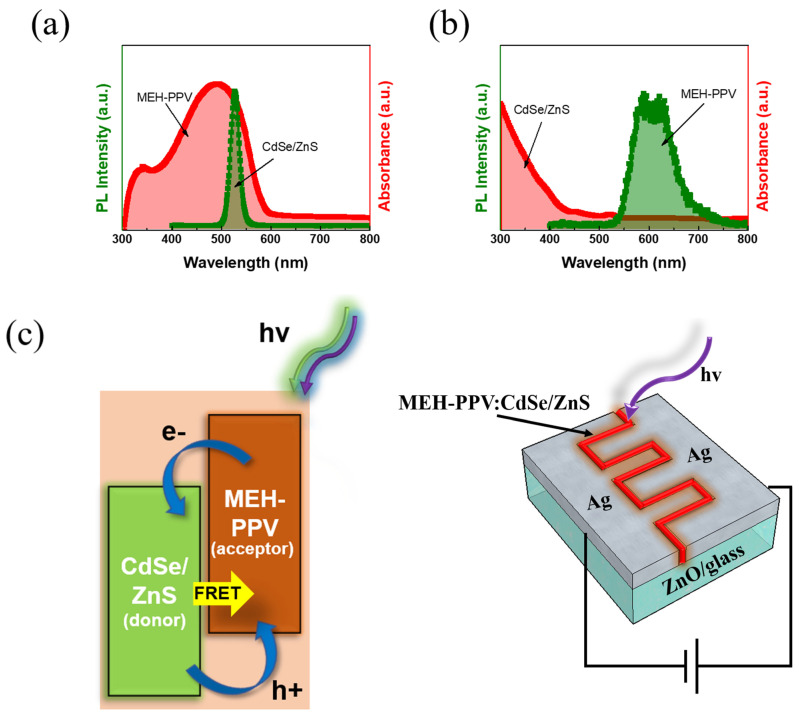
Overlap of spectral region between the (**a**) MEH−PPV absorbance spectrum and CdSe/ZnS CSQDs PL, (**b**) MEH−PPV PL and CdSe/ZnS CSQDs absorbance spectrum (QD concentration is 5%), and (**c**) schematic energy band diagram of the MEH−PPV:CdSe/ZnS composite and photosensor device structure. The mechanism of the MEH−PPV:CdSe/ZnS model is based on the FRET from CdSe/ZnS CSQDs to the MEH−PPV polymer molecules and the charge transfer process.

As seen in Figure 3a, the alignment of the PL spectrum of the CdSe/ZnS CSQDs with the absorbance spectrum of the MEH−PPV serves as compelling evidence of energy transfer from the QDs to MEH−PPV. This phenomenon is in line with the Föster mechanism [23,24,25,26,27,28]. There is a notable absence of a substantial overlap between the PL spectrum of MEH−PPV and the absorbance spectrum of QDs (Figure 3b). This absence indicates that the energy transfer process from the polymer to the QDs is insignificant. CdSe/ZnS functions as the donor, while the MEH−PPV polymer acts as the acceptor. Figures S3 and S4 (given in ESI) describe the dependence of the energy transfer dynamics on the QD size. There is no spectral overlap between the Red QD emission band and the MEH−PPV absorbance band (Figure S3). Meanwhile, the remarkable spectral overlap between the absorbance spectrum of MEH−PPV and the emission spectra of the Green QDs was observed clearly (Figure S4), affirming the efficient energy transfer between the QDs and polymer, thereby enhancing the device performance (Figure S5).

### 3.3. MEH−PPV:CdSe/ZnS Based Photodetector

#### 3.3.1. Photodetector Mechanism

A PD constructed using the MEH−PPV:CdSe/ZnS CSQDs composite was fabricated to facilitate further investigation. The operational mechanism of this device is illustrated in Figure 3c. The mechanism of the MEH−PPV:CdSe/ZnS model is based on Förster resonance energy transfer (FRET) from CdSe/ZnS QDs to MEH−PPV polymer molecules. It is well known that there are two expressions of energy transfer between organic polymers and inorganic nanoparticles based on Dexter and Förster’s theory [29]. FRET is a non-radiative interaction by which the donor can give its energy to the acceptor. It is thus often utilized in the case of composites comprising conjugated polymers and ligand-capped QDs [30,31,32]. In addition, the photosensor mechanism involving Ag/ZnO combined with MEH−PPV and CdSe/ZnS operates through a series of steps that enhance the photodetection capabilities across a broad spectral range. Upon illumination, ZnO and CdSe/ZnS QDs absorb photons from the UV spectrum, while MEH−PPV absorbs visible light photons, generating excitons in both components. The holes generated within the CdSe/ZnS QDs are transferred to the valence band of MEH−PPV, effectively separating the electron–hole pairs and preventing recombination within the QDs. At the interface between these materials, the electrons and holes are efficiently separated: electrons are directed towards the ZnO layer and subsequently collected by the Ag electrode, while holes are transported through MEH−PPV to the opposite electrode. This charge separation and transport process results in the generation of a photocurrent. The Ag electrode, in combination with ZnO, enhances electron collection due to the high conductivity of Ag and the excellent electron mobility of ZnO, leading to a lower recombination rate and stronger photocurrent. This integration results in a photosensor with improved sensitivity and efficiency that is capable of responding to a wide range of light wavelengths.

#### 3.3.2. Photodetector Performance

**Figure 4:**
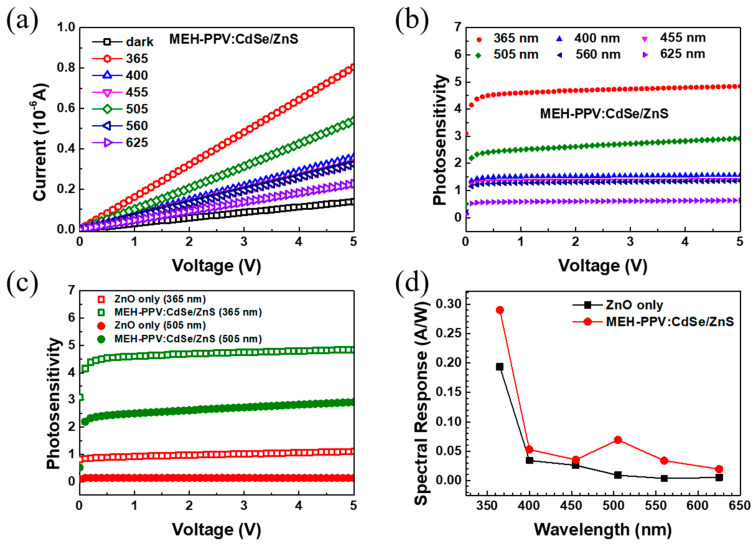
(**a**) Typical I-V curves under darkness and illumination, (**b**) photosensitivity of MEH−PPV:CdSe/ZnS-based PD, (**c**) comparison of photosensitivity of PDs based on ZnO only and MEH−PPV:CdSe/ZnS, and (**d**) comparison of response of PD based on ZnO only, MEH−PPV, CdSe/ZnS, and MEH−PPV:CdSe/ZnS at the bias of 5V. The PDs based on MEH−PPV, CdSe/ZnS, and MEH−PPV:CdSe/Zn were fabricated on ZnO/glass substrates. The concentration of CdSe/ZnS in the composite is 5%.

Figure S6 (given in ESI) explores the impact of various QD concentrations in polymer/QD composites on the performance of PDs, highlighting an optimal concentration of 5% (further details are available in ESI). The typical current–voltage (I-V) characteristics of PDs based on ZnO with/without MEH−PPV:CdSeZnS (5%) under dark and illuminated conditions are presented in Figure 4a. The photocurrent typically increases with increasing forward bias voltage due to the enhanced transport of charge carriers. Specifically, it was observed that the photocurrent generated under illumination at wavelengths of 365 nm and 505 nm exhibited a notable increase compared to other wavelengths. This increase in photocurrent at these specific wavelengths signifies the pronounced sensitivity of the photosensor to light in the UV and visible spectral regions. Figure 4b shows the photosensitivity of MEH−PPV:CdSe/ZnS-based PDs, as calculated by Equation (S2) (provided in ESI). Figure 4c shows a significant enhancement in the photosensitivity at wavelengths of 365 nm and 505 nm when using the MEH−PPV:CdSe/ZnS composite in the PD compared to the PD without it. Specifically, at 365 nm, the photosensitivity of the MEH−PPV:CdSe/ZnS-based PD increased approximately 5-fold, marking a remarkable 2.5-fold increase compared to the ZnO-only-based PD. Similarly, at 505 nm, the I_photo_/I_dark_ ratio of the MEH−PPV:CdSe/ZnS-based PD reached ~3-fold, while the ZnO-only-based PD exhibited no discernible response. This enhancement underscores the superior photosensitivity and detection capabilities conferred by the incorporation of the MEH−PPV:CdSe/ZnS composite, highlighting its efficacy in facilitating efficient charge generation and collection, particularly across UV and visible wavelengths. Figure 4d shows the comparison of the spectral response R (the calculated equation is supplied in ESI, Equation (S3) [33]) of PDs based on ZnO only, MEH−PPV, CdSe/ZnS, and MEH−PPV:CdSe/ZnS. The only ZnO-based PD displayed the spectral response ~0.2 A/W at a wavelength of 365 nm due to the absorbance in the UV range of ZnO. The CdSe/ZnS QD-based PD showed a response predominantly in the UV region. This is attributed to the QDs’ ability to absorb and respond to UV light effectively. In contrast, the MEH−PPV-based PD shows a strong response in the visible range. This is due to the significant absorbance of MEH−PPV in the visible spectrum, which enables it to convert visible light into an electrical signal efficiently. Meanwhile, with adding the MEH−PPV:CdSe mixture, the PD exhibited a wide spectral response region. Particularly noteworthy is its peak response, reaching values of ~0.3 A/W and ~0.07 A/W at wavelengths of 365 nm and 505 nm, respectively. In addition to the spectral response, the specific detectivity D* (the calculated equation is supplied in ESI, Equation (S4) [34]) was also enhanced noticeably in MEH−PPV:CdSe/ZnS PD (Figure S7). The ability to detect a broad spectrum is attributed to the advantageous properties of both ZnO and CdSe/ZnS, which exhibit high absorbance in the UV range, and MEH−PPV, known for its high absorbance in the visible range.

Table 1 shows a comparison of the performance of the PDs discussed in this work and previous reports. The MEH−PPV:CdSe/ZnS-based PD demonstrates superior responsivity in the UV range, particularly at a wavelength of 365 nm, when compared to PDs based on other materials. This enhanced performance is evident through a significant increase in the responsivity metrics, as reported in recent studies [35,36,37,38,39,40,41]. The integration of MEH−PPV with CdSe/ZnS quantum dots optimizes the photodetector’s ability to absorb UV light and convert it into an electrical signal more efficiently than other material combinations. This advancement underscores the potential of MEH−PPV:CdSe/ZnS-based PDs to be used in applications requiring high sensitivity in the UV spectrum, making them a preferable choice over traditional PDs that may employ materials like CdS, PbS, or other polymer–QD composites. 

## 4. Conclusions

In summary, this study investigated the Förster Resonance Energy Transfer phenomenon from CdSe/ZnS CSQDs to a MEH−PPV polymer. PDs based on ZnO utilizing the MEH−PPV:CdSe/ZnS nanocomposite were successfully fabricated, demonstrating outstanding photocurrent and photosensitivity in both the UV and visible wavelengths. The pivotal role of the FRET mechanism, facilitating energy transfer from CdSe/ZnS QDs to MEH−PPV, along with the combination of high absorbance materials in both the UV and visible wavelengths, contributed to the high performance of the wide-spectra PD devices. 

## Figures and Tables

**Table 1:** Comparison of the performance of ZnO-based PDs.

Material	Wavelength (nm)	Bias (V)	Responsivity (A/W)	References
ZnO/PbS QDs	370	30	6.332 × 10^−3^	[35]
N–C QDs/ZnO	365	−8	96.2 × 10^−3^	[36]
ZnO@CdS/PEDOS	UV—Visible	0	7.68 × 10^−3^ (365 nm) 2.53 × 10^−3^ (450 nm)	[37]
ZnO-MoS_2_	UV—Visible	5	0.03	[38]
PANI/ZnO MW	350	0	0.56 × 10^−3^	[39]
p-ZnO/n-Si	365	5	0.16	[40]
ZnO/PEDOT:PSS	380	−2	0.013	[41]
ZnO/MEH−PPV:CdSe/ZnS	UV—Visible	5	0.3 (365 nm) 0.07 (505 nm)	This work

## Data Availability

The original contributions presented in the study are included in the article/Supplementary Material, further inquiries can be directed to the corresponding author/s.

## Supplementary Materials

The following supporting information can be downloaded at: https://www.mdpi.com/article/10.3390/polym16162371/s1, Figure S1: (a) FE−TEM images and (b) Particle size distribution of CdSe/ZnS QDs; Figure S2: FE−SEM cross-section image of PD device; Figure S3: Overlap spectral region between (a) MEH−PPV absorbance spectrum and CdSe/ZnS CSQDs PL, (b) MEH−PPV PL and CdSe/ZnS CSQDs absorbance spectrum. The size of QD ~9 nm; Figure S4: Overlap spectral region between (a) MEH−PPV absorbance spectrum and CdSe/ZnS CSQDs PL, (b) MEH−PPV PL and CdSe/ZnS CSQDs absorbance spectrum. The size of QD ~6.5 nm; Figure S5: I-V curves in the dark and under illumination of PDs based on (a) MEH−PPV:CdSe/ZnS (QD size ~6.5 nm), and (b) MEH−PPV:CdSe/ZnS (QD size ~9 nm); and (c) Comparison of response of PDs without and with MEH−PPV:CdSe/ZnS (QD size ~6.5 nm and 9 nm, respectively) at the bias of 5V; Figure S6: I-V curves in the dark and under illumination (365 nm and 505 nm) of PDs based on (a) MEH−PPV:CdSe/ZnS (QD concentration ~3%), (b) MEH−PPV:CdSe/ZnS (QD concentration ~5%), (c) MEH−PPV:CdSe/ZnS (QD concentration ~10%), and (d) Comparison of spectral response of PDs based on composites with different concentration of QDs (3, 5, and 10%) at the bias of 5V; Figure S7: Comparison of detectivity of PDs without and with MEH−PPV:CdSe/ZnS; Table S1: Intensity of the light sources.
