# Supplementary material for: Expanding the Spectral Responsivity of Photodetectors via the Integration of CdSe/ZnS Quantum Dots and MEH−PPV Polymer Composite
Source: Polymers (Basel). 2024 Aug 21;16(16):2371. doi: 10.3390/polym16162371 (PMC11359818; doi:10.3390/polym16162371)
Supplement: Supplementary file 1 [file polymers-16-02371-s001.zip › polymers-3131552-supplementary.pdf]

Supplementary Materials

(a)

(b)

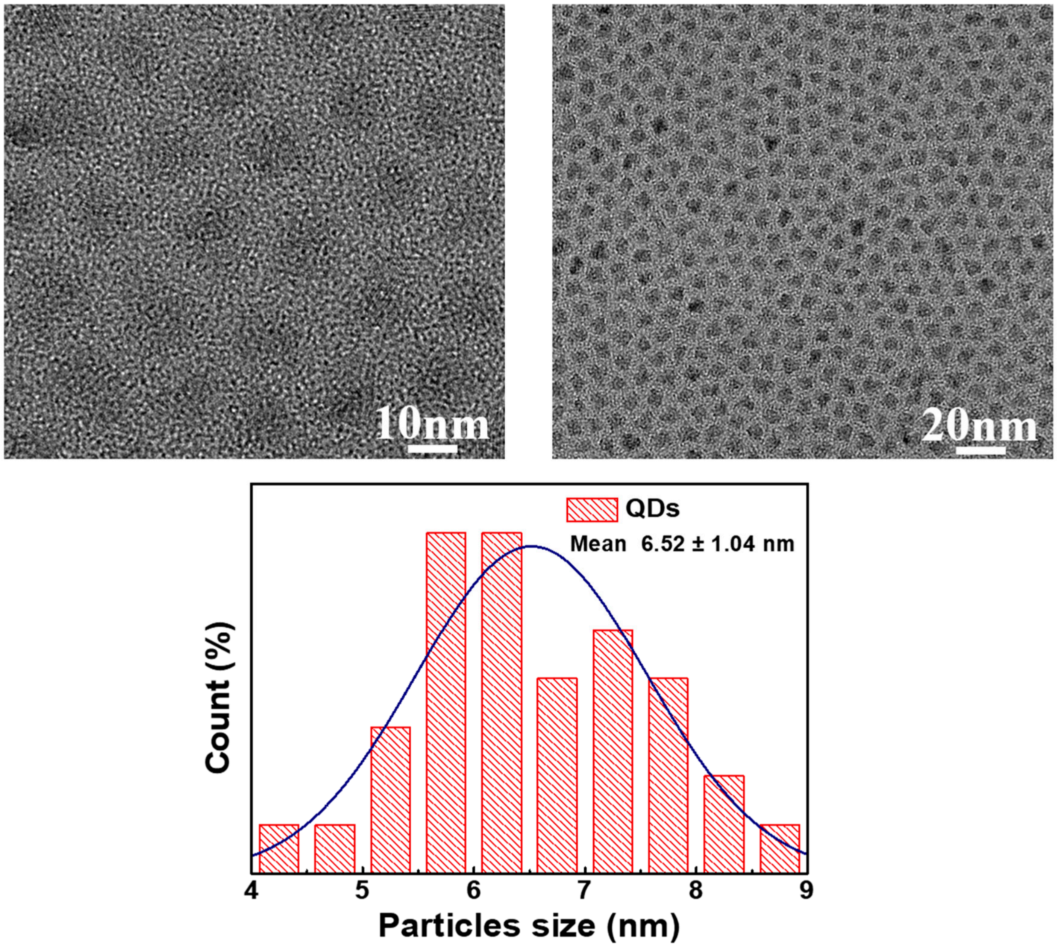

Figure S1: (a) FE-TEM images and (b) Particle size distribution of CdSe/ZnS QDs

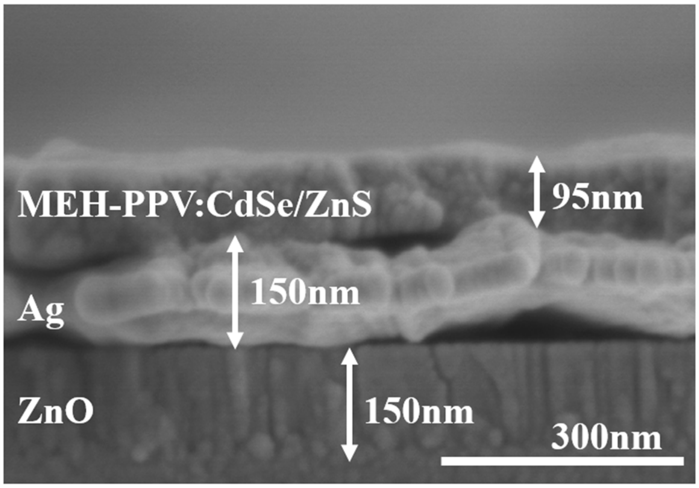

Figure S2: FE-SEM cross-section image of PD device

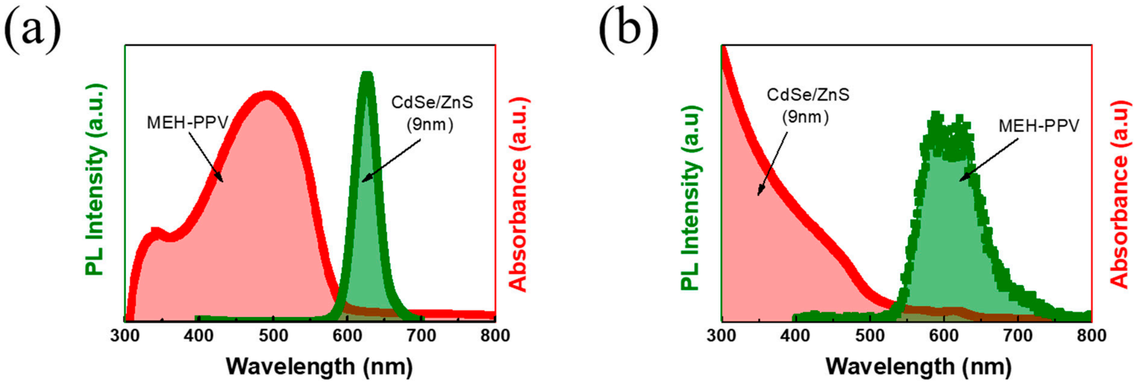

Figure S3: Overlap spectral region between (a) MEH-PPV absorbance spectrum and CdSe/ZnS CSQDs PL, (b) MEH-PPV PL and CdSe/ZnS CSQDs absorbance spectrum. The size of QD ~ 9nm

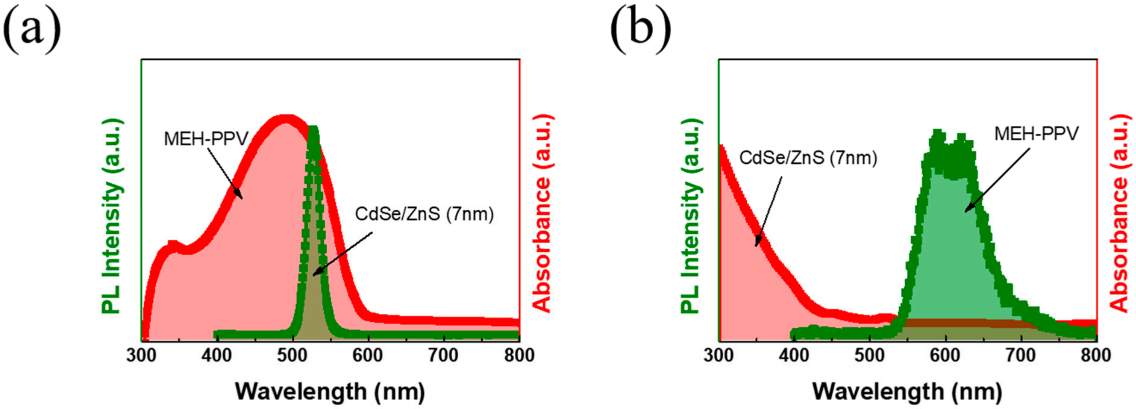

Figure S4: Overlap spectral region between (a) MEH-PPV absorbance spectrum and CdSe/ZnS CSQDs PL, (b) MEH-PPV PL and CdSe/ZnS CSQDs absorbance spectrum. The size of QD ~ 6.5nm

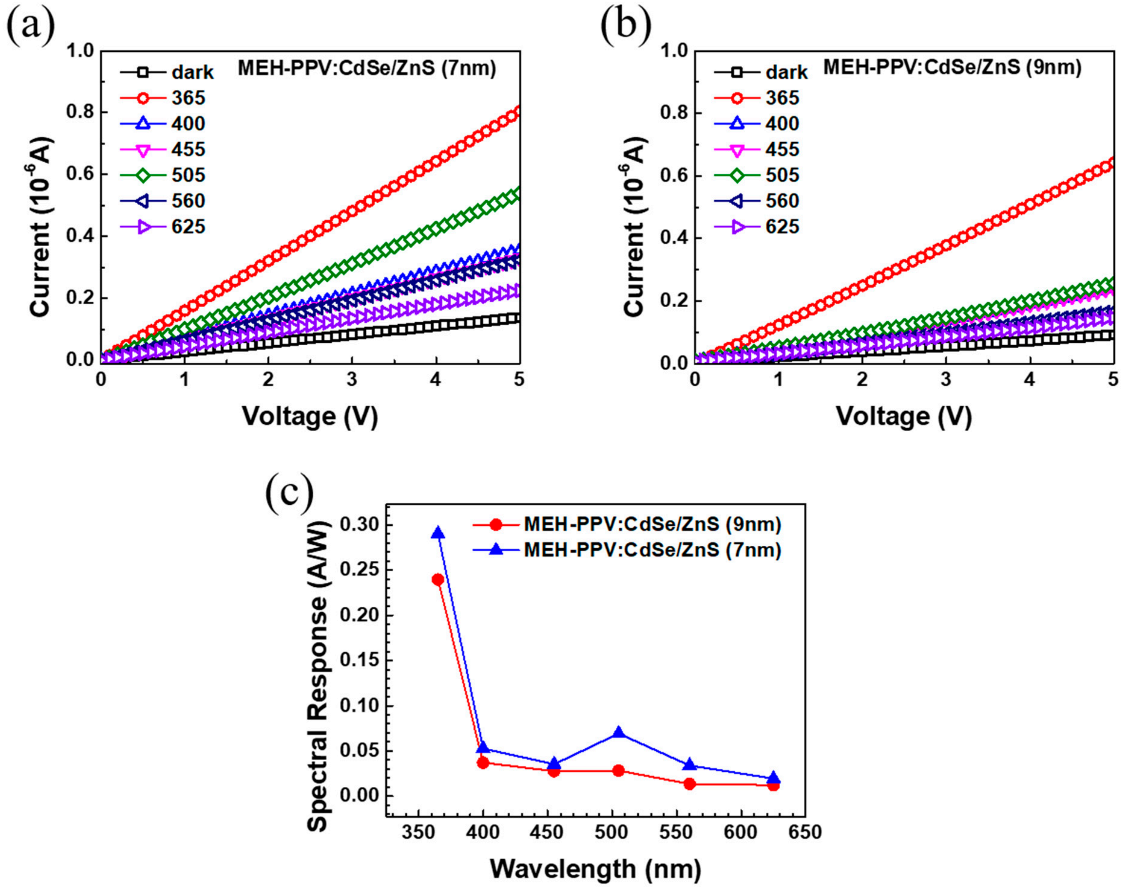

Figure S5: I-V curves in the dark and under illumination of PDs based on (a) MEH-PPV:CdSe/ZnS (QD size ~ 6.5nm), and (b) MEH-PPV:CdSe/ZnS (QD size ~ 9nm); and (c) Comparison of response of PDs without and with MEH-PPV:CdSe/ZnS (QD size ~ 6.5nm and 9nm, respectively) at the bias of 5V

We conducted experiments utilizing QDs of varying sizes (the concentration of QDs in composites is 5%), specifically 9nm and 6.5nm: The strength of the Coulomb interaction between electrons and holes within QDs influences charge transfer processes, such as exciton dissociation and recombination. In smaller QDs, the stronger Coulomb attraction can facilitate more efficient charge transfer, leading to enhanced photoconductivity and improved device performance in applications such as photodetectors. Coulomb interactions also play a role in energy transfer processes between QDs and neighboring materials. Hence, the size of CdSe/ZnS QDs affects energy transfer processes in composite materials. For instance, in QD-polymer composites, the size of the QDs determines the efficiency of energy transfer between the QDs and the polymer matrix. In Figure S2, it's evident that with the 9nm QDs, there's no spectral overlap between the absorbance of MEH-PPV (a polymer) and the

photoluminescence (PL) of CdSe/ZnS QDs, nor between the absorbance of CdSe/ZnS QDs and the PL of MEH-PPV. This absence of overlap indicates that there's no energy transfer occurring between the QDs and the polymer, and vice versa. However, in Figure S3, when using 6.5nm QDs, there's a clear overlap between the absorbance of MEH-PPV and the PL of CdSe/ZnS QDs. This overlap signifies that there is indeed an energy transfer from the QDs to the polymer. Thus, CdSe/ZnS play a role of donor and MEH-PPV is an acceptor. Consequently, the efficiency of the composite of QDs and polymer is enhanced with 6.5nm QDs. Further substantiating this observation, in Figure S4, the performance of the photodetectors (PDs) fabricated with 6.5nm QDs is notably superior to those made with 9nm QDs. This confirms that utilizing QDs with a size of ~ 6.5nm yields better results in terms of performance. Based on these findings, we have concluded that employing QDs with a size of 6.5nm is optimal for our study.

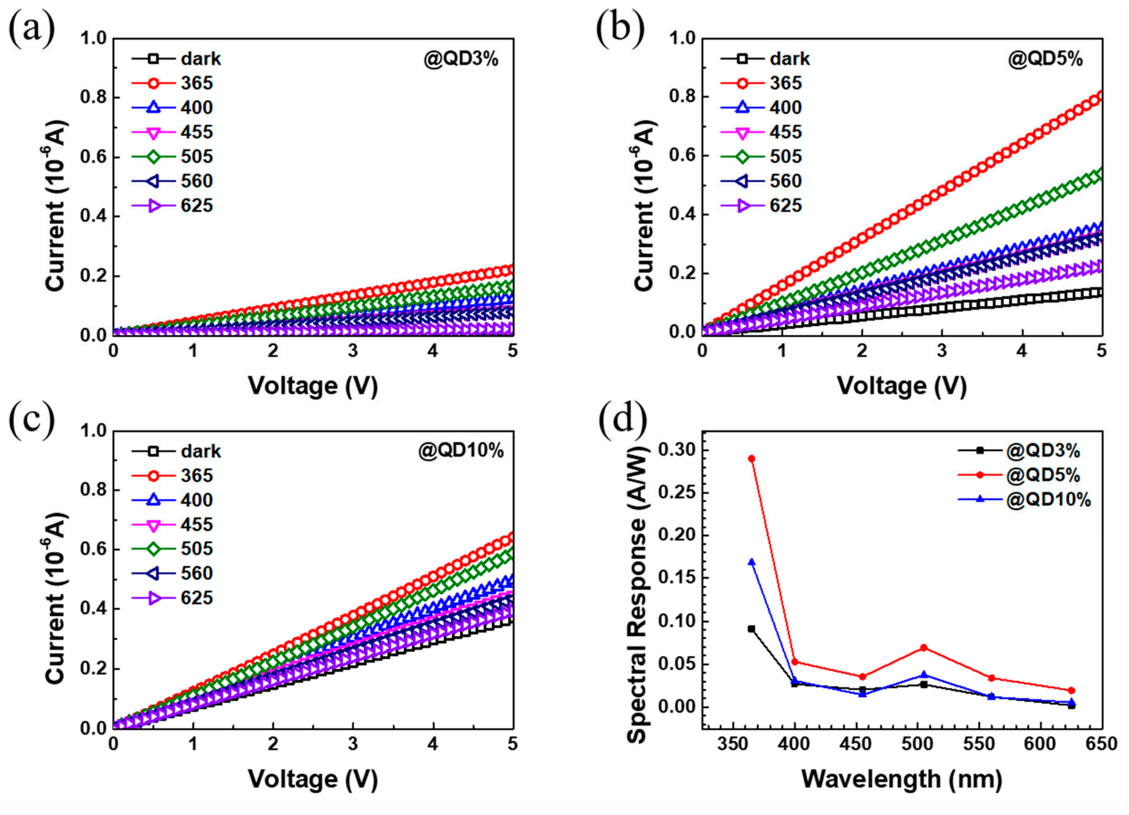

Figure S6: I-V curves in the dark and under illumination (365 nm and 505 nm) of PDs based on (a) MEH-PPV:CdSe/ZnS (QD concentration ~3%), (b) MEH-PPV:CdSe/ZnS (QD concentration ~5%), (c) MEH-PPV:CdSe/ZnS (QD concentration ~10%), and (d) Comparison of spectral response of PDs based on composites with different concentration of QDs (3, 5, and 10%) at the bias of 5V

We designed the experiment with QD concentration at 3, 5, and 10%. The photocurrent increases remarkably when we increase the concentration of QDs in composite from 3 to 5%. When the concentration of QDs is too low (3%), the efficiency of energy transfer from the QDs to the polymer may be compromised. This is because a low concentration of QDs means fewer available QDs to absorb photons and generate excitons. As a result, there may not be enough excitons to effectively transfer energy to the polymer, leading to reduced overall efficiency in the device. Notably, at QDs concentration of ~10%, the photocurrent experienced a notable decrease, consequently diminishing the overall performance of the sensor (Figure S4). This reduction in photocurrent suggests that excessively high QD concentrations may lead to undesirable effects such as increased charge carrier recombination or inter-QD interactions that impede charge transport efficiency. Therefore, the chosen 5% QD concentration strikes a balance, maximizing light absorption and charge carrier generation while minimizing detrimental effects on device performance, thereby ensuring optimal sensor functionality and sensitivity.

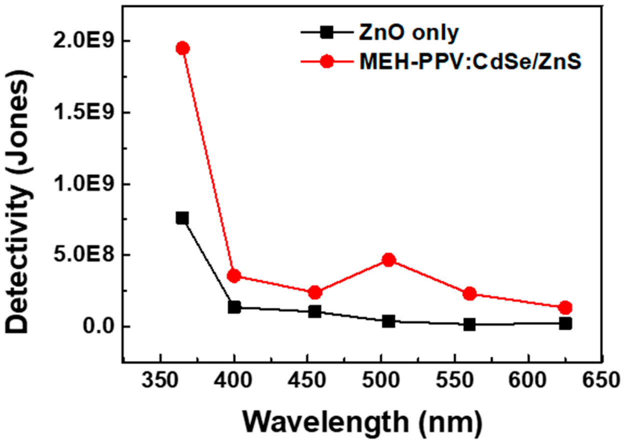

Figure S7: Comparison of detectivity of PDs without and with MEH-PPV:CdSe/ZnS

Table S1 Intensity of light sources

Wavelength (nm)	365	400	455	505	560	625
Intensity (10^{-6} W)	2.3	4.1	5.4	5.8	5.5	4.5

For direct bandgap materials, the relationship between optical bandgap and photon energy is given as

$$\alpha h\nu = A(h\nu - E_g)^{\frac{1}{2}} \qquad \text{(Eq. S1)}$$

Photosensitivity can be calculated by the formula:

$$\text{Sensitivity}_{photo} = (I_{photo}-I_{dark})/I_{dark} \quad \text{(Eq. S2)}$$

The spectral response R can be calculated by:

$$R = \frac{Iphoto-Idark}{P}, \quad \text{(Eq. S3)}$$

where P is power of incident light.

Besides responsivity, the specific detectivity (D*) is also one of the most important parameters and it characterizes how weak light photosensor can detect. When the dark current is dominated by short noise, D* can be expressed as

$$D^* = \frac{A^{\frac{1}{2}}R}{(2qI_{dark})^{\frac{1}{2}}} \text{ ,} \quad . \quad \text{(Eq. S4)}$$

where A is the photodetector area, and q is the charge of electron.
